# The Ultimate Poker Face: A Case Report of Facial Diplegia, a Guillain-Barré Variant

**DOI:** 10.5811/cpcem.2020.2.45556

**Published:** 2020-04-23

**Authors:** Joshua Lowe, James Pfaff

**Affiliations:** Brooke Army Medical Center, Department of Emergency Medicine, Fort Sam Houston, Texas

**Keywords:** Facial Diplegia, Guillain-Barré variant

## Abstract

**Introduction:**

Facial diplegia, a rare variant of Guillain-Barré syndrome (GBS), is a challenging diagnosis to make in the emergency department due to its resemblance to neurologic Lyme disease.

**Case report:**

We present a case of a 27-year-old previously healthy man who presented with bilateral facial paralysis.

**Discussion:**

Despite the variance in presentation, the recommended standard of practice for diagnostics (cerebrospinal fluid albumin-cytological dissociation) and disposition (admission for observation, intravenous immunoglobulin, and serial negative inspiratory force) of facial diplegia are the same as for other presentations of GBS.

**Conclusion:**

When presented with bilateral facial palsy emergency providers should consider autoimmune, infectious, idiopathic, metabolic, neoplastic, neurologic, and traumatic etiologies in addition to the much more common neurologic Lyme disease.

## INTRODUCTION

Facial diplegia is a rare variant of Guillain-Barré syndrome (GBS) where patients present with bilateral facial paralysis and paresthesia. Patients usually present 1–28 days after onset of symptoms, occasionally with extremity paresthesia but often in isolation.^2^ This variant occurs in less than 1% of all GBS patients.^1^–^2^ The pathophysiology of the disease is due to the acute inflammatory demyelinating polyneuropathy subtype much more often than the acute motor axonal neuropathy subtype of GBS.^2^ Due to its resemblance to the far more prevalent neurologic Lyme disease, it can be a potential pitfall diagnosis for emergency providers.

## CASE REPORT

A 27-year-old male military recruit without significant previous medical history was transported to the emergency department (ED) by ambulance with a chief complaint of bilateral facial paralysis. A resident of Puerto Rico, the patient had recently traveled to San Antonio, Texas, for military exercises and received multiple vaccines five days prior to onset of symptoms. He began experiencing mild paresthesia in his hands and feet four days prior to ED presentation. He reported progressive neurologic signs and symptoms including being unable to close his eyes or mouth, which made sleeping and eating difficult, as well as right-sided facial numbness. He went to the military medical clinic two days prior to ED presentation where he was diagnosed with a complex migraine and discharged to the barracks on quarters (strict bed rest) with ibuprofen and ondansetron and instructions to return for follow-up the next day to evaluate for resolution of symptoms.

During the subsequent ED visit his vital signs included a heart rate of 113 beats per minute, blood pressure 117/76 millimeters of mercury, respiration 16 breaths per minute, and temperature 98.5 degrees Fahrenheit. His blood glucose was 116 milligrams per deciliter (mg/dL) (90–120 mg/dL). Physical exam revealed symmetric bilateral facial paralysis, causing difficulty in closing his eyelids and mouth, which resulted in injected conjunctiva with moderate tearing, and cheilitis (Image). He endorsed decreased soft-touch sensation in a radial distribution in his upper extremities and the distribution of his fifth lumbar nerve in his lower extremities. Bilateral Achilles reflexes were absent. Strength and reflexes were otherwise preserved in his lower extremities. He was administered one liter of Ringer’s lactate, and the tachycardia improved.

Computed tomography of his head showed no masses or evidence of bleeding. Neurology was consulted and per their recommendation we obtained magnetic resonance imaging of his brain, which demonstrated no lesions, masses, or evidence of edema. A lumbar puncture was performed; it revealed albumin-cytological dissociation of cerebrospinal fluid (CSF), which is an increased protein with normal cell counts. Polymerase chain reaction did not detect any common bacterial or viral meningitis organisms. His complete blood count, comprehensive metabolic panel, and erythrocyte sedimentation rate were all within normal limits. The patient was given 10 milligrams dexamethasone intravenously in the ED, and blood cultures were obtained prior to admission.

**CPC-EM Capsule**

What do we already know about this clinical entity?

The treatment and disposition for all variants of Guillain-Barré syndrome (GBS) is the same.

What makes this presentation of disease reportable?

Facial diplegia is a rare variant of GBS that mimics a fairly common disease process in Lyme disease

What is the major learning point?

This case demonstrates the differential diagnosis and diagnostic pitfalls associated with facial diplegia.

How might this improve emergency medicine practice?

Clinicians should consider a thorough differential diagnosis for facial diplegia.

With a presumptive diagnosis of GBS the patient was admitted to the hospital for serial negative inspiratory flow measurements and intravenous immune globulin (IVIG), while awaiting further diagnostic testing by neurology. Serum and CSF studies detected none of the following organisms: *Borrelia burgdorferi*, Zika virus, West Nile virus, varicella-zoster virus, *Treponema pallidum*, human immunodeficiency virus, or *Mycobacterium tuberculosis*. Based upon the exam findings and diagnostic testing, the diagnosis of facial diplegia (GBS variant) was confirmed with the etiology most likely post-vaccinal. The patient received four days of IVIG and was discharged from the hospital. He returned to training with 4/5 strength and decreased light touch in his bilateral face and complete resolution of the paresthesia in his extremities. He was started on long-term daily corticosteroids by the neurologist with continued 4/5 strength and light touch in his bilateral face noted on his one month follow-up.

## DISCUSSION

Facial diplegia is a rare disorder, accounting for less than 2% of all patients with facial paralysis.^1^ The differential of facial diplegia includes Lyme disease, meningitis, and trauma. While Lyme disease is the most common etiology, GBS accounts for about 5% of cases (Table).^1^–^3^ Although facial nerve paralysis is involved in 27–50% of GBS cases (50% of those patients having bilateral involvement), facial diplegia is a rare presentation, occurring in 0.25–0.8% of all GBS patients. Causes are thought to be the same as in other variants of GBS.^2^,^4^

Common features of GBS include the following: subjective peripheral paresthesia (88%); a preceding illness one to two weeks prior to symptoms (75%); and CSF albumin-cytological dissociation (88%).^4^ The diagnostic criteria include core features of facial weakness, absence of ophthalmoplegia or truncal/cervical ataxia, and monophasic disease course with 12 hours to 28 days between onset and nadir. Supportive features include antecedent infectious symptoms, presence of distal paresthesia prior to facial diplegia, electrophysiological evidence of neuropathy, and CSF albumin-cytological dissociation.^5^

The prognosis for the facial diplegia subtype of GBS is generally favorable, as diaphragmatic paralysis has never been documented.^2^ Corticosteroids, although classically given, have not been shown to be effective in GBS.^2^ Although there have been no GBS-variant specific trials of treatment that show IVIG efficacy, the recommended standard of practice is still admission for observation, IVIG, and serial negative inspiratory force.^2^

## CONCLUSION

Facial diplegia is a rare variant of GBS that presents a very challenging diagnosis to make in the ED due to its wide differential diagnosis. When presented with bilateral facial palsy emergency providers should consider autoimmune, infectious, idiopathic, metabolic, neoplastic, neurologic, and traumatic etiologies in addition to the much more common neurologic Lyme disease. Facial diplegia is a clinical diagnosis that is bolstered by the presence of albumin-cytological dissociation in the cerebral spinal fluid.^2^ Treatment is the same as for other variants of GBS: observation; serial negative inspiratory force; and IVIG if the patient’s condition begins to deteriorate.

## Figures and Tables

**Image:**
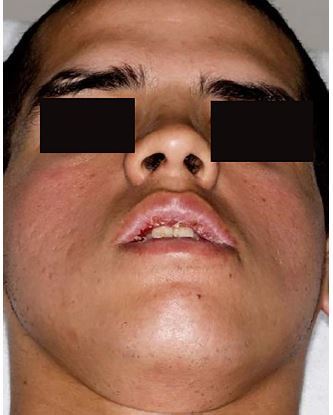
Patient with facial diplegia attempting to both wrinkle forehead and smile during neurologic exam.

**Table:** Differential diagnosis of acute onset bilateral facial paralysis.

Category	Diagnosis
Autoimmune	Guillain-Barré syndrome
	Sarcoidosis
	Myasthenia gravis
	Amyloidosis
Infectious	
	Lyme disease
	Herpes simplex virus
	Varicella-zoster virus
	Human immunodeficiency virus
	Syphilis
	Human T-cell leukemia virus-1
	Poliomyelitis
	Influenza
	Cytomegalovirus
	Botulism
	Epstein-Barr virus
Idiopathic	
	Idiopathic intracranial hypertension
	Bell’s palsy
Metabolic	
	Diabetes
Neoplastic	
	Leukemia
	Porphyria
	Meningioma
Neurological	
	Multiple sclerosis
Trauma	
	Skull fracture
	Parotid surgery
	Mastoid surgery
